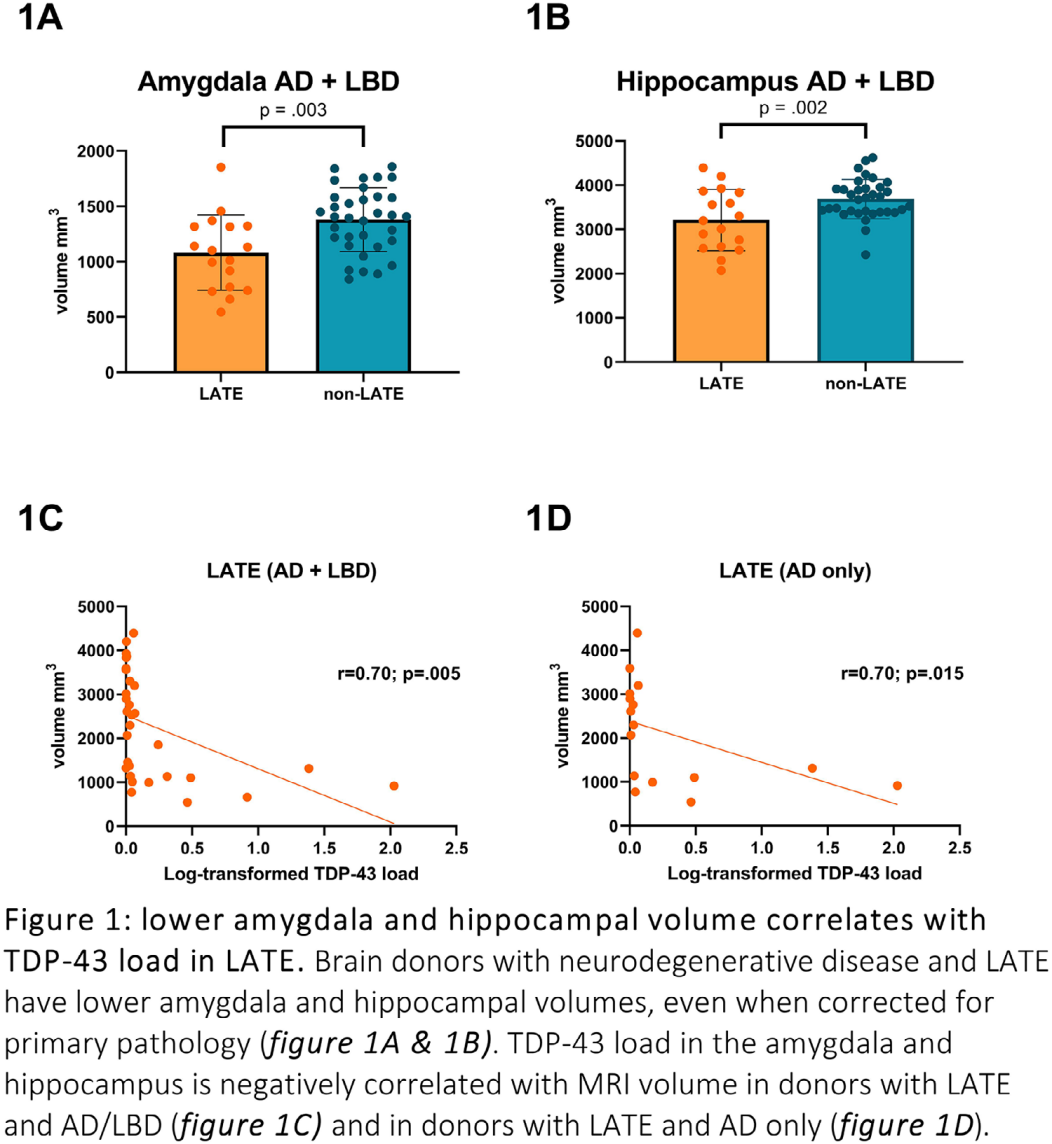# Lower amygdala and hippocampal volume correlates with TDP‐43 load in limbic‐predominant age‐related TDP‐43 encephalopathy (LATE)

**DOI:** 10.1002/alz.087361

**Published:** 2025-01-09

**Authors:** Alex J. Wesseling, Ismael Luis Calandri, Rik Ossenkoppele, Maud M.A. Bouwman, Natasja A. C. Deshayes, Wilma D.J. van de Berg, Annemieke J.M. Rozemuller, Yolande A.L. Pijnenburg, Jeroen J.M. Hoozemans, Laura E. Jonkman

**Affiliations:** ^1^ Amsterdam UMC, location VUmc, the Netherlands, Amsterdam Netherlands; ^2^ Amsterdam Neuroscience, Neurodegeneration, Amsterdam Netherlands; ^3^ Alzheimer Center Amsterdam, Neurology, Amsterdam UMC, Amsterdam Netherlands; ^4^ Alzheimer Center Amsterdam, Amsterdam UMC, Amsterdam Netherlands; ^5^ Amsterdam Neuroscience, Brain Imaging, Amsterdam Netherlands; ^6^ Amsterdam Neuroscience, Neurodegeneration, Vrije Universiteit Amsterdam, Amsterdam Netherlands; ^7^ Alzheimer Center Amsterdam, Neurology, Vrije Universiteit Amsterdam, Amsterdam UMC location VUmc, Amsterdam Netherlands; ^8^ Amsterdam UMC, location VUmc, Department of Anatomy and Neurosciences, Section Clinical Neuroanatomy and Biobanking, Amsterdam Netherlands; ^9^ Department of anatomy and neurosciences, Amsterdam University Medical Centers, Amsterdam Netherlands; ^10^ Amsterdam Neuroscience, brain imaging, Amsterdam Netherlands; ^11^ Department of Pathology, Amsterdam Neuroscience, Amsterdam UMC, Amsterdam, Netherlands, Amsterdam, Netherlands, Amsterdam Netherlands; ^12^ Alzheimer Center, Department of Neurology, Amsterdam UMC, Vrije Universiteit Amsterdam, Amsterdam Neuroscience, Amsterdam Netherlands; ^13^ Amsterdam Neuroscience, Neurodegeneration, Amsterdam, Noord‐Holland Netherlands

## Abstract

**Background:**

Limbic‐predominant age‐related TDP‐43 encephalopathy (LATE) is often described as occurring in older individuals, either with or without co‐occurring neurodegenerative disease. Because the presence of LATE can only be determined post mortem, little is known about the clinical and neuroimaging features of LATE. The current study aims to assess the correlation between LATE and MRI‐measured amygdala and hippocampal volume in Alzheimer’s disease (AD) and Lewy Body Diseases (LBD), including Parkinson’s disease (PD), PD dementia, and dementia with Lewy Bodies

**Methods:**

Post‐mortem in‐situ 3DT1 3T‐MRI data were collected for 51 cases (27 AD and 24 LBD) of which 17 had post‐mortem confirmed LATE neuropathological change (9 AD and 8 LBD), as well as 34 non‐LATE (18 AD and 16 PD) donors (matched on age, sex, and Braak stage). Right hemisphere amygdala and hippocampal volumes were calculated using Freesurfer. Pathological load of TDP‐43, p‐tau and α‐synuclein from the same regions were obtained using IHC and quantified using QuPath. Group differences were assessed with univariate analyses and correlations using linear mixed models, both using age, sex, post‐mortem delay and intracranial volume as covariates.

**Results:**

Two AD cases with a disease onset below 65 showed LATE pathology. In the combined cohort, brain donors with LATE neuropathological change showed significantly lower amygdala (p=.003) and hippocampal (p=.002) volumes, even when corrected for p‐tau and Lewy body load (Figure 1a). In AD, this difference remained (amygdala p=.001, hippocampus p=.007, corrected for p‐tau), but not in the LBD group. In donors with LATE, TDP‐43 load correlated negatively with MRI measured amygdala and hippocampal volume when corrected for p‐tau and Lewy body load (r= .70; p=.005) (Figure 1b). The same association was found in AD (r=.70; p=.015 corrected for tau load) but not in LBD donors.

**Conclusion:**

These results suggest that TDP‐43 inclusions plays a role in hippocampal and amygdala atrophy on MRI, even when correcting for effects of primary pathology. Moreover, a salient detail is that LATE also seems to play a role in the disease presentation of young‐onset dementia patients.